# Yak genome database: a multi-omics analysis platform

**DOI:** 10.1186/s12864-024-10274-6

**Published:** 2024-04-05

**Authors:** Hui Jiang, Zhi-Xin Chai, Xiao-Ying Chen, Cheng-Fu Zhang, Yong Zhu, Qiu-Mei Ji, Jin-Wei Xin

**Affiliations:** 1 State Key Laboratory of Hulless Barley and Yak Germplasm Resources and Genetic Improvement, 850000 Lhasa, Tibet China; 2 Institute of Animal Science and Veterinary, Tibet Academy of Agricultural and Animal Husbandry Sciences, 850000 Lhasa, Tibet China; 3 Key Laboratory of Qinghai-Tibetan Plateau Animal Genetic Resource Reservation and Utilization, Sichuan Province and Ministry of Education, Southwest Minzu University, 610041 Chengdu, Sichuan China

**Keywords:** Yak, Genome, Database, Multi-omics, Plateau environment

## Abstract

**Background:**

The yak (*Bos grunniens*) is a large ruminant species that lives in high-altitude regions and exhibits excellent adaptation to the plateau environments. To further understand the genetic characteristics and adaptive mechanisms of yak, we have developed a multi-omics database of yak including genome, transcriptome, proteome, and DNA methylation data.

**Description:**

The Yak Genome Database (http://yakgenomics.com/) integrates the research results of genome, transcriptome, proteome, and DNA methylation, and provides an integrated platform for researchers to share and exchange omics data. The database contains 26,518 genes, 62 transcriptomes, 144,309 proteome spectra, and 22,478 methylation sites of yak. The genome module provides access to yak genome sequences, gene annotations and variant information. The transcriptome module offers transcriptome data from various tissues of yak and cattle strains at different developmental stages. The proteome module presents protein profiles from diverse yak organs. Additionally, the DNA methylation module shows the DNA methylation information at each base of the whole genome. Functions of data downloading and browsing, functional gene exploration, and experimental practice were available for the database.

**Conclusion:**

This comprehensive database provides a valuable resource for further investigations on development, molecular mechanisms underlying high-altitude adaptation, and molecular breeding of yak.

## Background

Although single omics study provides information and insights into specific biological or molecular processes, it is hard to confirm the real molecular mechanisms underlying the functionality of an organism and the relationships between biological processes and environmental factors. Integrating and analyzing multiple omics data provide an effective and systematic approach to life science researchers. In general, genomics provides DNA sequence information, transcriptomics examines gene transcription patterns under specific conditions, proteomics explores the composition and expression levels of proteins in cells, and DNA methylation involves chemical modifications on DNA molecules [1]. Multi-omics analysis combines data at different levels to comprehensively explore biological processes. Multi-omics analysis reveals connections between genomics, transcriptomics, proteomics, and DNA methylation data, facilitating to understand how genomic variations impact gene transcription and protein expression, as well as the associations between DNA methylation and gene activities [2]. These pieces of information contribute novel information to the gene regulatory networks, which are important to molecular mechanisms underlying biological functions, development, metabolism, etiopathology, and environmental adaptation.

The yak (*Bos grunniens*) is a unique species in the Qinghai-Tibet Plateau, and widely distributes in high-altitude areas of Western China and neighboring regions. As a large mammal at the highest-altitude area, yak has survived and adapted to the harsh and cold environment after thousands of years of evolution [3]. Their unique biological features make them an ideal model for studying adaptive evolution and high-altitude ecosystems. Yak also plays important roles in agriculture and economic development. As a significant livestock species, yak provides meat, fur, and other economic resources. Their dung is also an important source of agricultural fertilizer and energy production. Moreover, yak positively impacts the ecological balance and vegetation restoration in the plateau grasslands through their grazing behaviors [4]. In recent years, we have analyzed yak using different omics approaches. These data preliminarily explored the yak genetic characteristics, gene transcription, protein expression, and DNA methylation patterns, as well as molecular regulatory mechanisms in response to different conditions [5–11], providing novel insights into the mechanisms underlying evolution, and high-altitude adaptation in yak.

Currently, the data resources of yak omics researches are generally stored in public databases in their raw data format, such as NCBI. These databases primarily provide storage and retrieval functions, but lack an integrated platform for data integration and in-depth analysis. Hu et al. [12] developed a yak genome database (http://me.lzu.edu.cn/yak), which incorporated genome sequences, predicted genes and associated annotations, non-coding RNA sequences, transposable elements, and single nucleotide variants of yak, as well as three-way whole-genome alignments between human, cattle and yak. However, this database did not include other omics datasets, such as transcriptome, proteome, and DNA methylation. Given the vast and diverse nature of omics data, the traditional database retrieval methods could not fully explore the relationship between different types of datasets [13]. Thus, an integrated platform of different omics data is crucial to facilitate data integration, interaction, and analysis. An integrated platform can also offer advanced data mining and machine learning algorithms to help researchers discover the complex relationships among yak genomics, transcriptomics, proteomics, and other omics levels, further deepening our understanding of biological processes and diseases in yak.

In this study, the Yak Genome Database (http://yakgenomics.com/) was constructed, which successfully assembled a comprehensive yak fine-scale genome map at the chromosome level, using PacBio sequencing, Illumina sequencing, Bionano assembly, and Hi-C three-dimensional genome scaffolding. Moreover, this platform also integrated transcriptome, proteome, and DNA methylation data of yak, which were not available in Yak Genome Database developed by Hu et al. [12]. This database provides basic information for yak researches in future, such as molecular breeding, molecular evolution, disease prevention and control.

## Construction and content

The Yak Genome Database was deployed in the Ubuntu 20.04 operation system using the AKKA 2.13 (web server), MySQL 8.0.30 (database server), Scala 2.13.2, and SBT 1.3.9. All data were managed and stored using the MySQL Database Management System. The query function was enforced based on Slick 3.3.2 middleware tier. The Jbrowse 1.16.11 was used to visualize the genome. The website interfaces were designed and implemented using the Bootstrap 4.6.0 and the Play Framework 2.8.7. The software versions and statistical tools used for data analyses and plot preparation have been presented in Xin et al. [6–11]. The boxplots, and heatmaps were prepared using R 4.2.1. The website has been tested in several popular web browsers, including Firefox, Google Chrome, and Internet Explorer.

## Utility and discussion

### The yak genome database content

**Fig. 1:**
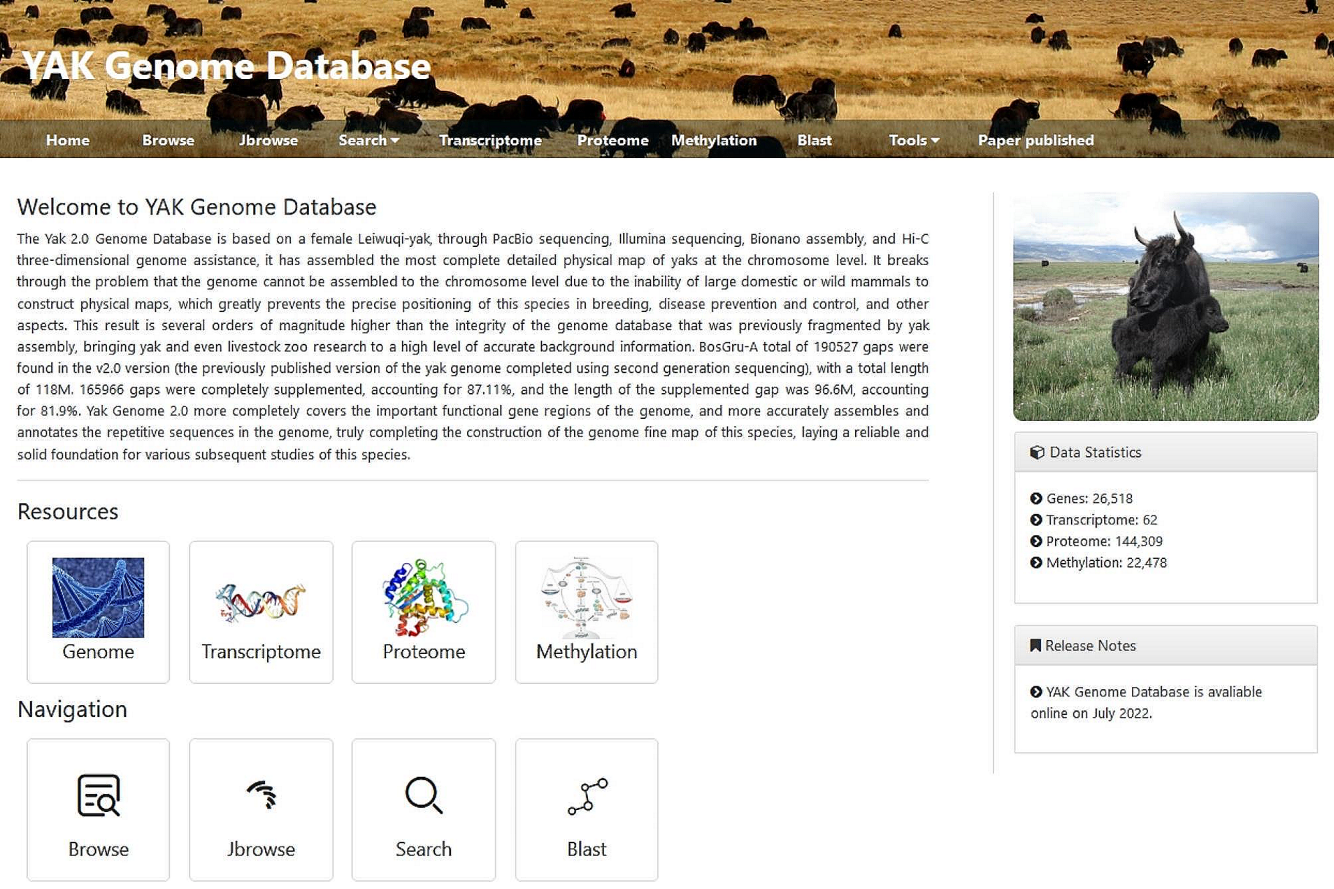
The homepage of yak genome database

The multi-omics data in the Yak Genome Database are categorized into two central functional domains: data resources and navigation (Fig. 1). The data resources contain four main modules, including genome, transcriptome, proteome, and methylation information. The database contains 26,518 genes, 62 transcriptomes, 144,309 proteome spectra, and 22,478 methylation sites of yak. The navigation page consists of Browser, Jbrowse, Search and Blast functions. Currently, the database supports individual download of images and gene data. In the future, we will add functions such as one-click download of whole genome information.

### Genome module

The Genome module incorporates the complete genomic DNA sequence of yaks obtained by the third-generation high-throughput sequencing platform (PacBio RSII) [14]. The yak genome was sequenced at a coverage of 70X, with the second-generation sequencing data used to correct errors. The Bionano assisted assembly technology was used for high-quality assembly, and analysis. Next, a refined physical map of the yak chromosome was generated, providing a more readable and complete genome database than the fragmented information in another Yak Genome Database (BosGru_v2.0) [15], and contributing a novel genome tool to yak researchers.

**Fig. 2:**
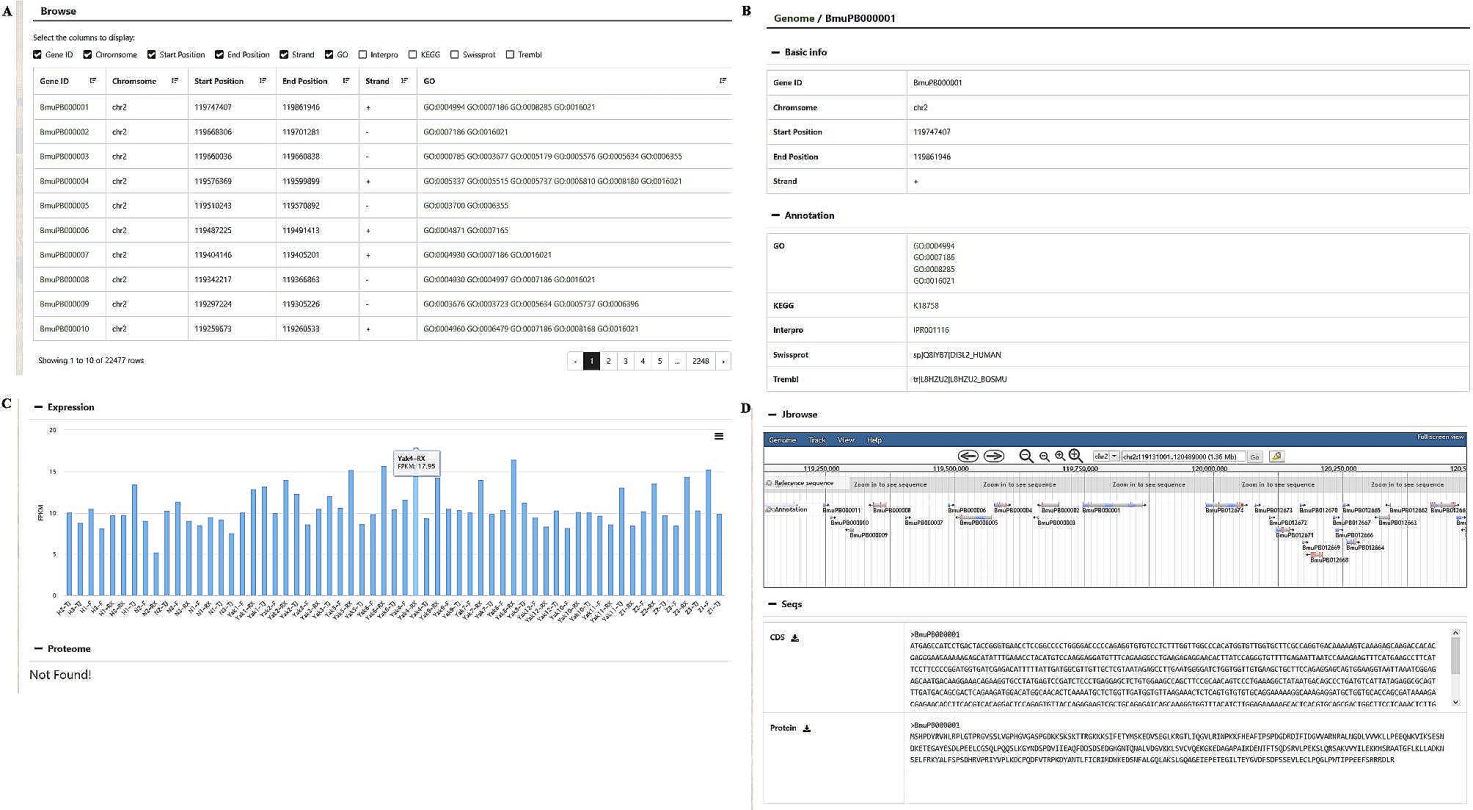
Features of the genome module. (**A**) Genome browse. (**B**) Basic information and annotation of a gene. (**C**) Gene expression. (**D**) Gene Jbrowse and sequences

When accessing the ‘Genome’ section on the homepage, a new page will display information of genes at all locations, such as Gene ID, Chromosome, Start Position, End Position, Strand, GO (Gene Ontology) terms, Interpro, KEGG (Kyoto Encyclopedia of genes and Genomes), Swissprot, and Trembl in a user-friendly table format (Fig. 2A). When clicking each gene, users can access detailed information of this gene, including annotations, transcriptional levels, proteome data, Jbrowse page, and nucleotide sequences associated with the gene (Fig. 2B-2D). The ‘Annotation’ tav provides comprehensive gene annotation information, including GO terms, KEGG pathways, and Interpro annotations, which can be further explored by clicking them. The ‘Expression’ tab displays gene expression levels across different cattle breeds and tissues, and users can download the images in various formats by selecting the menu in the upper right corner of the image. ‘Jbrowse’ is used to display integrated information from annotated genomic datasets, while ‘Seqs’ provides the coding sequence (CDS) and protein sequence on the selected gene.

### Transcriptome module

**Fig. 3:**
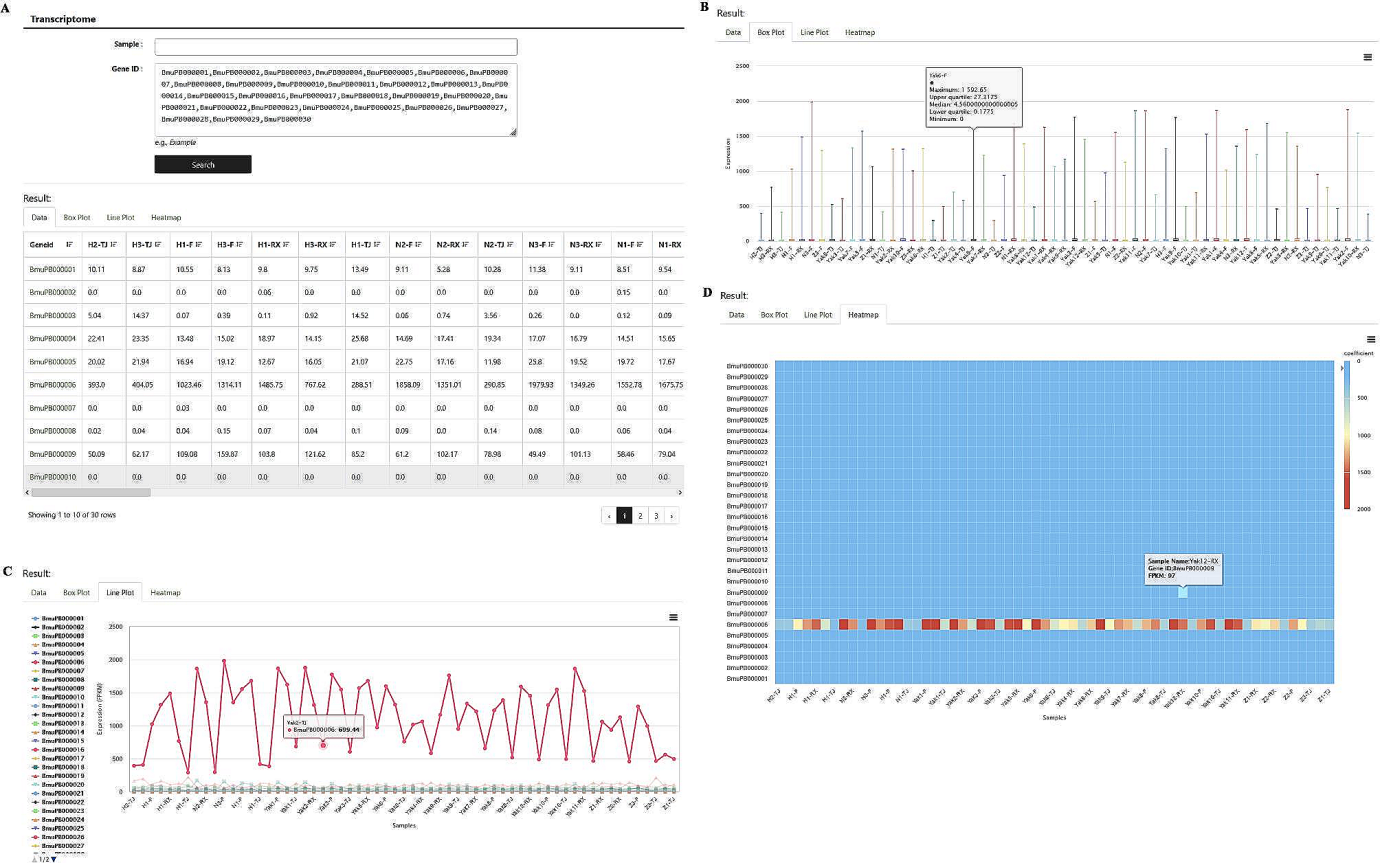
Features of the transcriptome module. (**A**) Transcriptome browse. (**B**) Box plot, (**C**) Line plot and (**D**) Heatmap of gene expression

Previously, comparative transcriptome sequencing was performed on lung, gluteal muscle, and mammary gland tissues of low-altitude cattle (Sanjiang and Holstein cattle), Tibetan cattle (living at a moderate altitude), and yaks (living at a high altitude). In addition, these tissues of yaks at different ages (6, 30, 60, and 90 months) were also subjected to transcriptome sequencing. These analyses identified the functional genes involved in the major biochemical, metabolic, and signal transduction pathways involved in yak development and high-altitude adaptation [10, 11]. These data are included in the transcriptome module on the website, providing a valuable transcriptome database for specific tissue biomarkers, molecular research, and breeding of yaks. After clicking the “Transcriptome” button, users can select the strain in the ‘Sample’ dialog box, enter the gene ID in the ‘Gene ID’ dialog box, and then click ‘Search’ (Fig. 3A), and then the website will return the transcriptional levels of the selected genes in selected samples in the forms of data table, Boxplot, Lineplot, and Heatmap (Fig. 3B and D).

### Proteome module

**Fig. 4:**
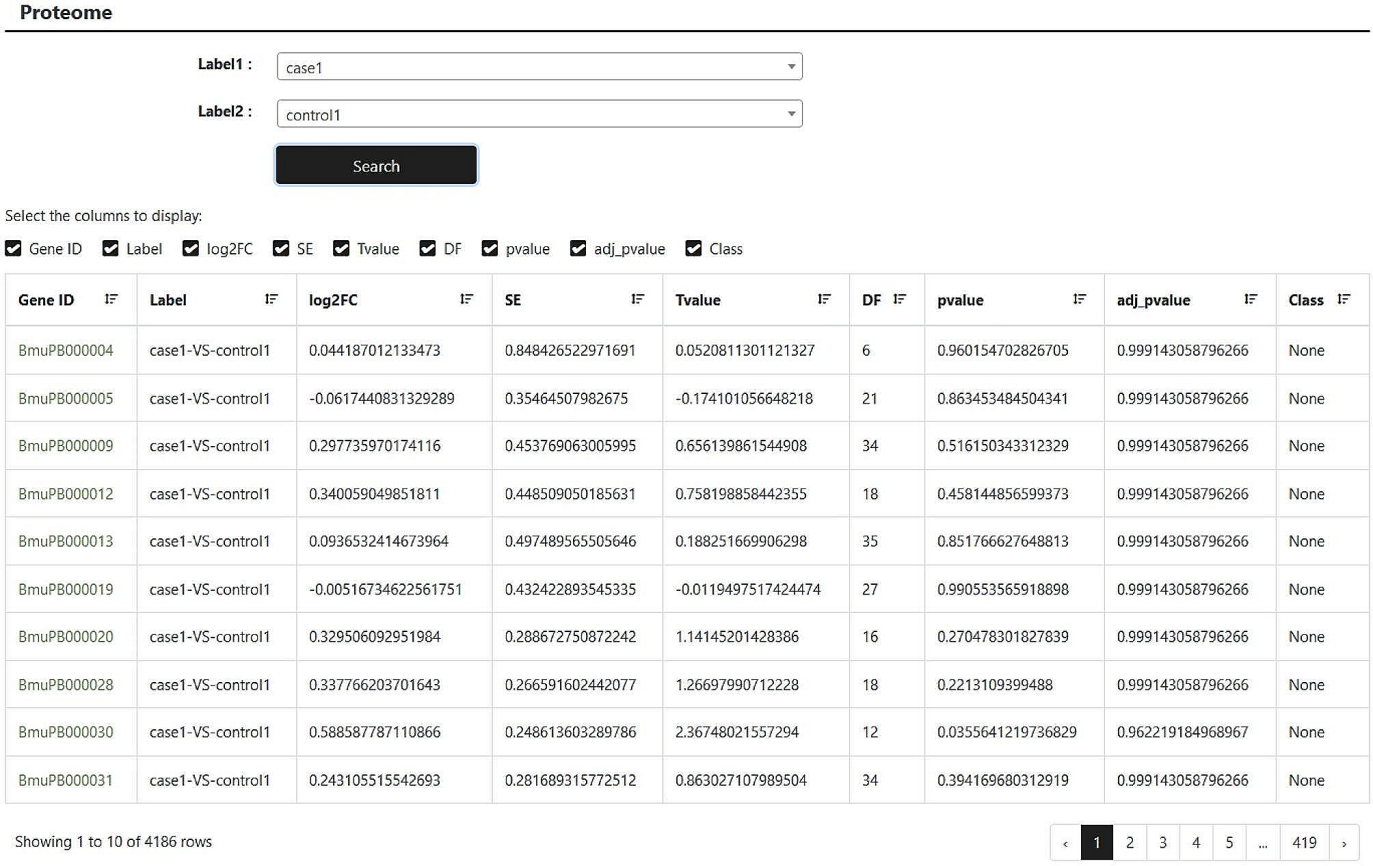
Browse of the proteome module

Using the liquid chromatography-mass spectrometry (LC-MS) method, proteomic analyses were conducted for four specific tissues from four different species (yak, Tibetan cattle, Sanjiang cattle, and Holstein cattle) [7–9]. All the animals were female and 60 months of age. The proteome module provides two input dialog boxes. Users can select two samples and then click the “search” button. Next, the website will return the comparison results of the expression levels of all genes in the two selected samples, including log2(fold change) and statistical parameters (Fig. 4).

### Methylation module

**Fig. 5:**
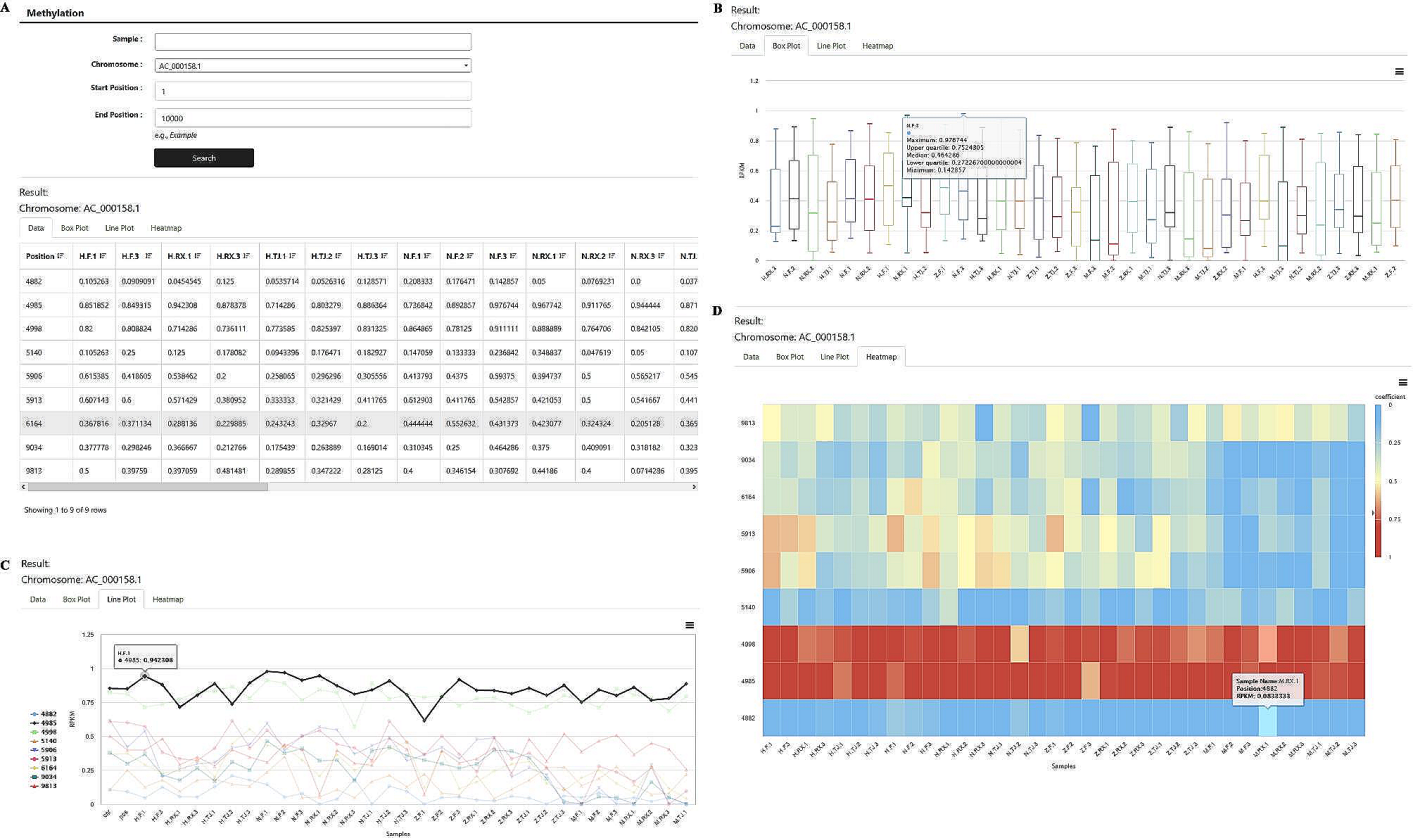
Features of the methylation module. (**B**) Box plot, (**C**) Line plot and (**D**) Heatmap of methylated gene expression

DNA methylation is a critical epigenetic modification that occurs in both animals and plants, playing pivotal roles in chromosome structure, gene expression and regulation [16]. The establishment of a comprehensive DNA methylation database for yak can significantly advance the comprehension of cellular gene expression and regulation, and provide deeper insights into the spatiotemporal specificity of DNA methylation across various developmental stages and organs [17]. The DNA methylation database of yak presents single-base methylation maps and tissue-specific methylation maps. The single-base methylation maps include: 1) DNA methylation levels at the single-base resolution, 2) DNA methylation levels specific to different base types, 3) DNA methylation levels specific to different gene structures, 4) DNA methylation levels in repetitive sequences, and 5) DNA methylation levels in non-coding sequences and regulatory regions. The tissue-specific methylation maps involve three tissues: mammary gland, lung, and muscle [6]. On the website, users can select ‘Sample’ and ‘Chromosome’ in the Methylation module, set the ‘Start Position’ and ‘End Position,’ and finally click ‘Search’ to obtain the corresponding DNA methylation results on the selected sequences (Fig. 5).

### Navigation

**Fig. 6:**
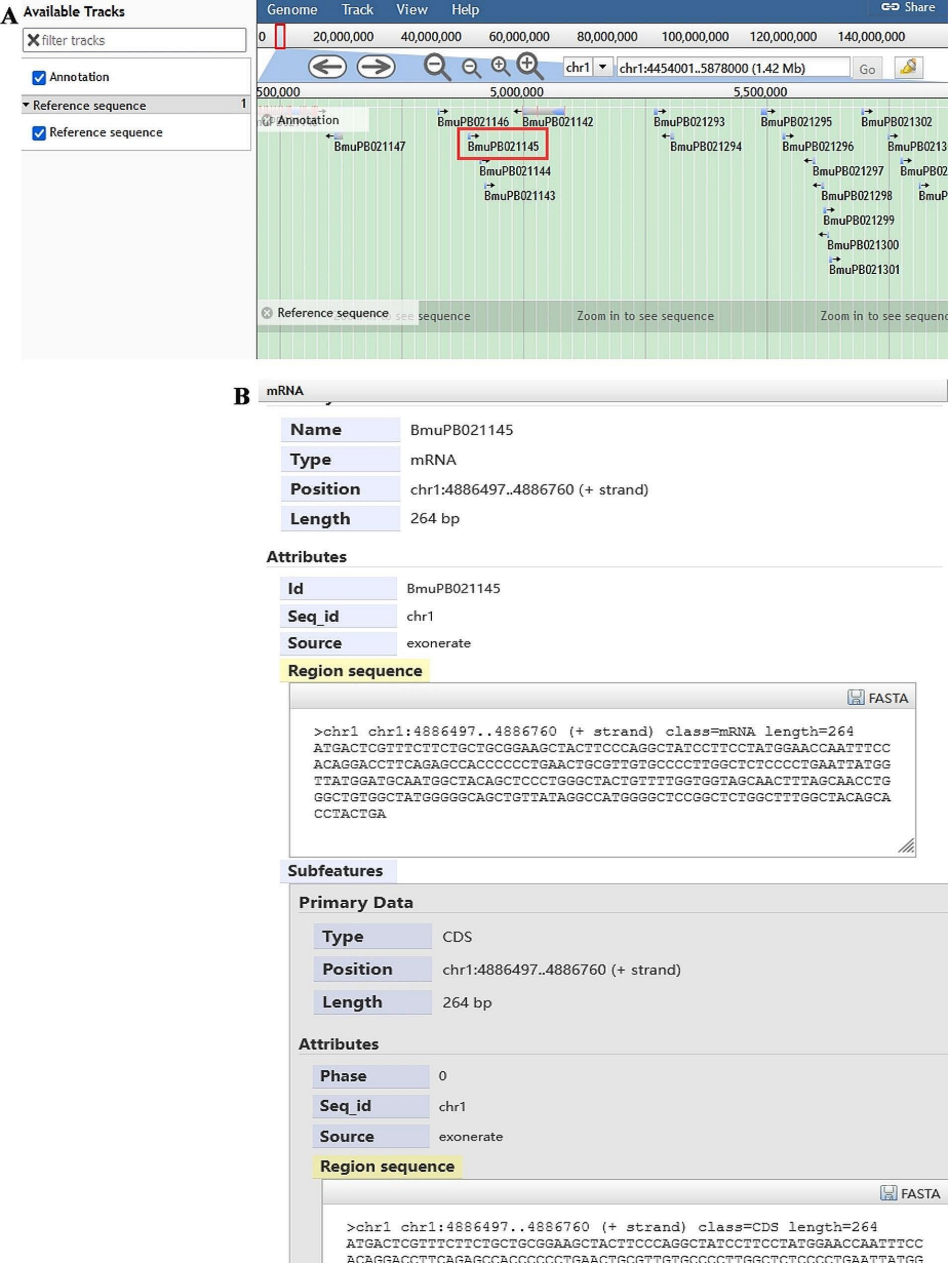
Regional view of the genome using Jbrowse. (**A**) A graphic view of the region 4,454,001 bp to 5,878,000 bp on Chr1. (**B**) The interface after clicking on ‘BmuPB021145’.

‘Browse’ allows users to read the yak genome directly. ‘JBrowse’ is a next-generation genome browser built with JavaScript and HTML5. The Jbrowse of Yak Genome Database includes tracks describing gene, gene sequence, mRNAs, structure, and other gene-related features, and provides a graphical display of annotations on the yak genome (Fig. 6). Users can browse gene models on chromosomes and unanchored contigs. For example, if user set the genomic region from 4,454,001 bp to 5,878,000 bp on Chr1 for browsing, all genes in this region will appear in order (Fig. 6A). When clicking on ‘BmuPB021145’, an extra layer will appear with the detailed information, such as mRNAs, CDS and other features (Fig. 6B). For more operational details, users can click the ‘Help’ button, which provides comprehensive instructions and guidance.

The ‘Search’ tab supplies users with two methods (search by gene ID or range) for genome searching. When users click on ‘Blast’, three options ‘Blastn Gene’, ‘Blastn Genome’ and ‘Blastp’ will display. Users can select the Blast type and enter a DNA or protein sequence, and set the parameters of ‘Evalue’, ‘Word size’ and ‘Max target seqs’. After clicking the ‘Search’ button, the nucleotide or protein sequence complying the search conditions will display and could be downloaded by the users.

### Additional tools

The Yak Genome Database also provides users with several convenient online tools, including Primer designer, GO and KEGG enrichment. The ‘Primer designer’ tool offers primer design function to amplify a selected sequence. The ‘GO enrichment’ and ‘KEGG enrichment’ tools facilitate the users to obtain the GO and KEGG enrichment results of a set of genes.

### Maintenance of the yak genome database in future

To ensure continuous operation of the Yak Genome Database, we would assign an administrator to manage the website regularly. We would keep omics studies on yak in future, and all the omics data we obtained would be uploaded to this database. In addition, we would keep cooperations with other investigators and find more cooperators who work on yak. Next, all the progresses on yak omics would also be encouraged to supplement in this database.

## Conclusions

The Yak Genome Database is a comprehensive platform of genomic physical map, which integrates genome, transcriptome, proteome, and DNA methylation data. Information in the database can be downloaded, and shared through the Internet. Users who want to upload their own data can contact the administrator of the website. By providing timely updates on yak research progress, the Yak Genome Database enables efficient and interactive sharing of existing scientific data among researchers worldwide who are interested in yak, cattle, livestock, ruminant animals, and even medical research. Comparative analysis of multidimensional data from key yak tissues aims to uncover the mechanisms underlying high-altitude adaptation, disease resistance, cold tolerance, and starvation resistance of large animals in the plateau. These findings contribute to molecular breeding of livestock animals and the understanding of human responses to harsh environments.

## Data Availability

The datasets generated and analyzed in the current study are freely available on the Download page of Yak database with the web link: http://yakgenomics.com/.

## Abbreviations

CDS: Coding Sequence
GO: Gene Ontology
KEGG: Kyoto Encyclopedia of Genes and Genomes
NCBI: National Center for Biotechnology Information
